# T1BT* structural study of an anti-plasmodial peptide through NMR and molecular dynamics

**DOI:** 10.1186/1475-2875-12-104

**Published:** 2013-03-18

**Authors:** Elena Topchiy, Geoffrey S Armstrong, Katherine I Boswell, Ginka S Buchner, Jan Kubelka, Teresa E Lehmann

**Affiliations:** 1 Department of Chemistry, University of Wyoming Laramie, Laramie, WY 82071, USA; 2 Department of Chemistry and Biochemistry, University of Colorado at Boulder, Aurora, CO, 80045, USA

**Keywords:** Medicinal chemistry, NMR, Circular dichroism, Malaria

## Abstract

**Background:**

T1BT* is a peptide construct containing the T1 and B epitopes located in the 5’ minor repeat and the 3’ major repeat of the central repeat region of the *Plasmodium falciparum* circumsporozoite protein (CSP), respectively, and the universal T* epitope located in the C-terminus of the same protein. This peptide construct, with B = (NANP)_3_, has been found to elicit antisporozoite antibodies and gamma-interferon-screening T-cell responses in inbred strains of mice and in outbred nonhuman primates. On the other hand, NMR and CD spectroscopies have identified the peptide B’ = (NPNA)_3_ as the structural unit of the major repeat in the CSP, rather than the more commonly quoted NANP. With the goal of assessing the structural impact of the NPNA cadence on a proven anti-plasmodial peptide, the solution structures of T1BT* and T1B’T* were determined in this work.

**Methods:**

NMR spectroscopy and molecular dynamics calculations were used to determine the solution structures of T1BT* and T1B’T*. These structures were compared to determine the main differences and similarities between them.

**Results:**

Both peptides exhibit radically different structures, with the T1B’T* showing strong helical tendencies. NMR and CD data, in conjunction with molecular modelling, provide additional information about the topologies of T1BT* and T1B’T*. Knowing the peptide structures required to elicit the proper immunogenic response can help in the design of more effective, conformationally defined malaria vaccine candidates. If peptides derived from the CSP are required to have helical structures to interact efficiently with their corresponding antibodies, a vaccine based on the T1B’T* construct should show higher efficiency as a pre-erythrocyte vaccine that would prevent infection of hepatocytes by sporozoites.

## Background

Malaria is the most prevalent parasitic human disease. It claims the lives of more children worldwide than any other infectious disease [1] Malaria is caused by micro-organisms of the genus *Plasmodium* and is transmitted to humans by mosquitoes. Among other species, *Plasmodium falciparum* causes the highest levels of mortality and morbidity [2]. Currently used prevention methods include indoor residual spraying, vector control and mosquito nets. However, none of these methods fully allows the eradication of malaria worldwide among humans. With increasing global prevalence of malaria and emerging resistance of *P. falciparum* to drug treatment, the need for an efficient malaria vaccine is greater than ever. The use of synthetic peptides for immunization is a very attractive strategy for antigen delivery, since they are relatively easy to obtain in large quantities with high purity. The circumsporozoite protein (CSP), covering the membranes of mature sporozoites, exhibits high immunogenicity and plays a crucial role in hepatic cells invasion by malaria parasites. This protein has been considered as a useful target for peptide-derived anti-plasmodial vaccine developments [3]. The central repeat region of the CSP, conserved amongst the different *Plasmodium* species, consists of 37 repeat units of the NANP amino acid sequence and four repeat units having the NVDP sequence for the NF54/3D7 strain [4]. The immunodominant epitope of the infective form of *P. falciparum* is the tandemly repeating tetrapeptide NANP [5]. Synthetic peptides derived from the repeat region of *P. falciparum* CSP have proven to be able to block CSP interactions with hepatocytes, as well as invasion of HepG2 cells [6,7]. Peptides that correspond to the epitope region of the CSP have been extensively studied to understand their immunogenicity. A multi-antigenic peptide construct, T1BT*, containing the T1 epitope (DPNANPNVDPNANPNV) from the central region, the B-cell activating epitope (NANP)_3_ from the tandem repeat region, and the universal T* epitope (EYLNKIQSLSTEWSPCSVT) from the C-terminus of the CSP; was found to elicit antisporozoite antibodies and gamma interferon-screening T-cell responses comparable to more complex tetrabranched peptides in inbred strains of mice and in outbred nonhuman primates [8].

Peptide vaccines elicit a variety of antibodies. Only some of these antibodies may bind appropriately to the cognate sequence in the native protein or the pathogen, since short, flexible peptides in solution can afford a variety of conformation. The production of effective vaccines requires a strategy that involves rational design of the peptide immunogen. In previous investigation of the solution, conformations of various immunogenic peptides in water solution, ^1^H nuclear magnetic resonance (NMR) and circular dichroism (CD) spectroscopies have proven very useful in determining the conformational preferences of peptides for folded forms [9-12]. These investigations included peptides with sequences (NANP)_n_ and (NPNA)_n_ with n = 1, 2, and 3; derived from the central repeat region of the *P. falciparum* CSP. The data derived from these studies were consistent with the presence of turn-like structures stabilized by hydrogen bonds. Spectral differences between peptides with different cadences of the tandemly repeating unit indicated that a repeating structural motif is formed by the NPNA cadence, rather than the alternative NANP [13]. In another set of studies, a computer model of Ac-(NPNA)_5_-NH_2_ peptide showed a backbone conformation in which each NPNA motif adopts a helical β-turn conformation [14]. In the present study, the three-dimensional structures of T1BT* and its analogue T1B’T*, where the B-cell activating tandem repeat was modified to be B’ = (NPNA)_3_, were determined through ^1^H-NMR spectroscopy. In light of the results of the Dyson *et al.* structural investigation of (NANP)_3_ and (NPNA)_3_ peptides [13], the goal of the investigation discussed herein is to determine the structural impact of the NPNA cadence on a peptide that has shown some level of protection against *P. falciparum* malaria [8]. Since the NPNA tandem repeat is more structured than the NANP repeat, further comparative structural studies of synthetic peptide vaccines based on NPNA and NANP are required. The possibility to change structural preferences of the synthetic peptide, depending on the sequence of the tandem repeat, might be a key step to improve the immunogenicity of CSP-based vaccines.

## Methods

### Studied peptides

Peptides T1BT*,1, T1B’T*, 2; and T1, 3 (Biomatik USA, LLC) were investigated in this work. The amino acid sequences for these peptides are: Ac-(DPNANPNV)_2_(NANP)_3_(EYLNKIQSLSTEWSPCSVT)-NH_2_, Ac-(DPNANPNV)_2_(NPNA)_3_(EYLNKIQSLSTEWSPCSVT)-NH_2_, and Ac-(DPNANPNV)_2_-NH_2_, respectively. A single-labelled peptide with amino acid sequence: Ac-(DPNANPNV)_2_(NANPNA*NPNANP)(EYLNKIQNSLSTEWSPCSVT)-NH_2_, 1**’**, where A* denotes Ala-(2,3,3,3-D4) (Cambridge Isotope Laboratories, Inc); and double-labelled peptides with sequences Ac-(DPNA*NPNVDPNANPNV)(NANPNANPN*ANP)(EYLNKIQNSLSTEWSPCSVT)-NH_2_, 1**”**, and Ac-(DPNANPNVDPNA*NPNV)(NPN*ANPNANPNA)(EYLNKIQNSLSTEWSPCSVT)-NH_2_, 2**”**, (American Peptide Company), where A* and N* denote Ala-(2,3,3,3 – D4) and Asn-(2,3,3 – D3, 12N2), respectively, were used to facilitate NMR signal assignments.

### Single-labelled peptide synthesis

The single-labelled peptide was chemically synthesized on a Tribute-automated peptide synthesizer (Protein Technologies, Inc) using standard FMOC solid-phase techniques. The crude peptide was purified by reverse-phase HPLC.

### NMR and CD sample preparation

NMR samples for 1, 2, and 1’ were prepared from the synthetic peptides by dissolving 3 mg in 25% deuterated acetonitrile and 75% H_2_O, to a final volume of 650 μl `and a final concentration 1mM. NMR samples for 3 were prepared by dissolving 3 mg of the synthetic peptide in 650 μl of solvent (10%D_2_O/90%H_2_O), to a final concentration of 1mM. NMR samples for 1” and 2” were prepared by dissolving 3 mg of dry peptide in 650 μl of solvent (10%D_2_O/90%H_2_O), to afford a final concentration of 1mM. For CD studies, 20 μM samples were prepared by dissolving 37.5 mg of dry peptide in 1.46 ml of H_2_O.

### NMR data collection

NMR spectra were performed at 900 MHz in a Varian NMR DirectDrive system (Agilent Technologies, Inc, Santa Clara, CA, USA) equipped with a cryogenically cooled, salt tolerant, ^13^C enhanced, triple resonance probe; and at 600 MHz in a Bruker Avance III 600 (Bruker BioSpin Corp, Billerica, MA, USA) with a 5.0 mm multi-nuclear broad-band observe probe. All NMR spectra were collected at 278 K and referenced to HDO as the internal standard with spectral widths of 16 ppm. TOCSY and NOESY collected at 900 MHz spectra were initially recorded with mixing times in the ranges of 60–100 and 60–250 ms, respectively. The best results were achieved with 100 ms mixing time for TOCSY and 200–250 ms for NOESY. These spectra were acquired with 32 transients and a number of increments ranging from 600 to 900 over a 90 kHz spectral width. In the direct dimension 4,096 points were collected over a 14.7 kHz spectral width. TOCSY and NOESY collected at 600 MHz were recorded with 512 t_1_ points and 2048 complex points for each free induction decay. The number of scans per t_1_ point was usually 32. Water suppression for all samples was performed with the WATERGATE sequence incorporated into all the two-dimensional experiments. Suppression of the acetonitrile signal was accomplished through pre-saturation, along with CW decoupling of ^13^C. Processing and analysis of the two-dimensional NMR data were performed on an Intel Xeon computer using NMRPipe [15], NMRViewJ [16] and Topspin3.0 (Bruker BioSpin Corp, Billerica, MA, USA) software. Spectra were Fourier transformed using a Lorentzian-to-Gaussian weighting and phase shifted sine-bell window functions.

### CD spectra collection

CD spectra were recorded on a Jasco J-815 CD spectrophotometer, using a 1 mm path cell at room temperature. Each CD spectrum was an average of 16 scans. The CD instrument was equipped with a Peltier temperature control.

### Average structure calculations

All calculations were carried out with Discovery Studio 3.1 (Accelrys, San Diego, CA) on an Intel Xeon 5600 series. Information about NOE-connectivities was obtained from the NMR experiments. The size of the NOEs was classified as strong, medium or weak, based on the intensity of each signal. NOE-derived distance constraints were set at 1.9-3.0, 2.5-4, and 3.5-5Å for strong, medium, and weak NOEs, respectively. The distance-dependent dielectric constant algorithm was used with an implicit solvent dielectric constant of 80. Nonbonded van der Waals interaction was cut-off at 14 Å. Molecular dynamics calculations used the Leapfrog Verlet dynamics integrator with a 0.001 ps time step. All energy minimizations and charge assignments used the CHARMm force field. Constant temperature and volume (NVT) with Berendsen thermal coupling was used as the dynamics ensemble for non-periodic systems. Long-range electrostatics was treated with spherical cut-off. The starting extended peptide structures were first minimized by steepest descent method followed by conjugate gradient minimization to an rms gradient of <0.001. The distance constraints were then applied, and the minimization steps were repeated. The structures were heated and equilibrated over 10 ps from 5 to 1,000 K, with velocities assigned every 0.001 ps. No distance constraints were used in this first step in order to randomize the structures. Molecular dynamics was ran for 4 ps, with distance constraints applied with a force constant of 0.06 kcal mol^-1^Å^-1^. Next, the force constants were scaled to 120 kcal mol^-1^ Å ^-1^ over 7 ps in a series of 0.4 ps molecular dynamics runs. The system was allowed to evolve for 6 ps, and then cooled to 300 K over 7 ps. At this temperature, the force constants were reduced to their final values of 60 kcal mol^-1^ Å ^-1^ over 4 ps in a series of 0.4 ps molecular dynamics runs. The system was allowed to equilibrate for 5 ps, followed by a final 15 ps molecular dynamics run. The coordinates of the final 5 ps of the 15 ps molecular dynamics were averaged and minimized by 1,000 steps of steepest descent method followed by conjugate gradient minimization to a rms gradient of <0.01 with distance constraints set to 60 mol^-1^ Å^-1^. The SHAKE algorithm [17] was used to fix all bond lengths to hydrogen atoms.

## Results

### NMR

The sequential assignment of the signals in the spectra of 1, 2 and 3 was performed through the overlap of their TOCSY and NOESY spectra. Repeating units and amino acids duplication caused the overlap of these signals, which required the use of single and double-labelled versions of 1 and 2 to achieve complete assignments. The resonance assignments for all peptides are shown in Tables 1, 2 and 3.

**Table 1:** **Assignments of the **^**1**^**H-NMR signals for 1 (ppm)**

**Residue**	**NH**	**C**^**α**^**H**	**C**^**β**^**H**	**C**^**γ**^**H**	**C**^**δ**^**H**	**C**^**ε**^**H**
Ac	8.95	4.81				
D1	8.48	4.81	2.96, 2.85			
P2		3.90	2.38, 2.16	1.49	3.01, 2.83	
N3	8.48	5.07	2.82			
A4	7.87	4.23	1.02			
N5	8.59	5.12	2.93, 2.88			
P6		3.90	2.38, 2.16	1.49	3.01, 2.83	
N7	8.46	4.80	2.94, 2.81			
V8	8.10	4.16	2.00	0.98		
D9	8.43	4.34	3.05, 2.88			
P10		3.90	2.38, 2.16	1.49	3.01, 2.83	
N11	8.25	5.07	3.00, 2.86			
A12	7.91	4.40	1.50			
N13	8.53	4.79	2.95, 2.83			
P14		3.90	2.38, 2.16	1.49	3.01, 2.83	
N15	8.48	4.81	2.96, 2.85			
V16	7.95	4.20	1.59	1.10, 1.00		
N17	8.31	4.80	2.96			
A18	8.02	4.38	1.49			
N19	8.44	5.07	3.00			
P20		3.90	2.38, 2.16	1.49	3.01, 2.83	
N21	8.39	4.78	2.94, 2.81			
A22	7.95	4.36	1.49			
N23	8.35	4.82	3.01, 2.80			
P24		3.90	2.38, 2.16	1.49	3.01, 2.83	
N25	8.43	4.84	3.01			
A26	7.97	4.37	1.48			
N27	8.35	4.82	3.01, 2.80			
P28		3.90	2.38, 2.16	1.49	3.01, 2.83	
E29	8.33	4.59	3.06, 3.01			
Y30	8.48	5.07	2.96, 2.85			
L31	7.95	4.28	1.78	1.67, 1.65	1.00	
N32	8.43	4.68	2.93, 2.90			
K33	8.10	4.38	1.95, 1.89	1.77	1.57, 1.51	1.99, 1.89
I34	8.25	4.36	1.48	1.48	1.09	
Q35	7.95	4.20	3.25	3.15		
N36	8.31	4.80	2.96			
S37	8.30	4.52	4.04, 3.99			
L38	8.28	4.50	1.85, 1.82	1.73	1.09, 1.00	
S39	8.25	4.53	4.05, 3.99			
T40	8.15	4.42	1.48	1.32		
E41	8.33	4.59	3.06, 3.01			
W42	8.11	4.76	3.41, 3.31			
S43	8.39	4.32	4.02, 3.97			
P44		3.55	2.38, 2.16	1.49	3.01, 2.83	
S46	8.30	4.52	4.04, 3.99			
V47	8.10	4.46	1.29	0.99		
T48	7.95	4.56	3.16	1.10		

Description: The data provided represents the assignments of the proton signals derivated form 1.

**Table 2:** **Assignments of the **^**1**^**H-NMR signals for 2 (ppm)**

**Residue**	**NH**	**C**^**α**^**H**	**C**^**β**^**H**	**C**^**γ**^**H**	**C**^**δ**^**H**	**C**^**ε**^**H**
Ac	8.82	4.78				
D1	8.37	4.80	3.05, 3.02			
P2		3.81	2.37, 2.03	2.01	2.81, 2.78	
N3	8.43	4.81	3.04			
A4	8.03	4.23	1.46			
N5	8.41	4.77	2.96, 2.88			
P6		3.81	2.37, 2.03	2.01	2.81, 2.78	
N7	8.41	4.77	2.96, 2.88			
V8	8.05	4.11	1.96	0.95		
D9	8.39	4.29	3.02, 2.88			
P10		3.87	2.38, 2.11	2.05	2.99, 2.61	
N11	8.45	4.75	2.91, 2.81			
A12	7.92	4.34	1.47			
N13	8.37	5.08	2.98			
P14		3.75	2.38, 2.10	2.05	2.95	
N15	8.33	4.75	2.93, 2.92			
V16	7.86	4.19	2.18	0.99		
N17	8.54	5.07	2.97, 2.82			
P18		3.50	2.38, 2.11	2.01	2.52, 2.51	
N19	8.22	4.74	2.92, 2.81			
A20	8.05	4.35	1.46			
N21	8.35	5.04	2.92, 2.77			
P22		3.51	2.38, 2.10	2.03	2.58	
N23	8.36	4.75	2.93, 2.91			
A24	8.01	4.35	1.46			
N25	8.41	4.81	2.96, 2.88			
P26		3.47	2.39, 2.10	2.01	2.52	
N27	8.18	4.74	2.92, 2.79			
A28	7.97	4.34	1.46			
E29	8.27	4.26	2.76, 2.75	2.91, 2.90		
Y30	8.05	4.53	3.21, 3.11			
L31	8.01	4.21	1.78, 1.76	1.63	1.01, 1.00	
N32	8.27	4.64	2.93, 2.77			
K33	8.11	4.30	1.69, 1.68	1.61	1.48, 1.45	1.79, 1.77
I34	8.25	4.32	1.30	1.05	1.01	
Q35	7.85	4.61	3.15, 3.11	3.05		
N36	8.43	4.82	3.04			
S37	8.39	4.54	3.93			
L38	8.25	4.46	1.81	1.69	1.02	
S39	8.26	4.49	4.02, 3.97			
T40	8.15	4.64	2.92	1.45		
E41	8.33	4.56	2.82, 2.81	3.01, 2.98		
W42	8.16	4.56	3.37, 3.28			
S43	8.24	4.50	4.02, 3.96			
P44		3.91	2.38, 2.11	2.06	2.85, 2.82	
C45	8.16	4.73	3.38, 3.28			
S46	8.24	4.50	4.02, 3.96			
V47	8.08	4.43	1.27	1.05, 1.04		
T48	8.21	4.64	2.01	1.29		

Description: The data provided represents the assignments of the proton signals derivated form 2.

**Table 3:** **Assignments of the **^**1**^**H-NMR signals for 3 (ppm)**

**Residue**	**NH**	**C**^**α**^**H**	**C**^**β**^**H**	**C**^**γ**^**H**	**C**^**δ**^**H**
Ac	8.93	4.63			
D1	8.47	4.63			
P2		4.34	2.26, 2.20	1.95, 1.90	3.71, 3.69
N3	8.42	4.66	2.79, 2.66		
A4	7.73	4.17	1.33		
N5	8.42	4.75	2.72, 2.54		
P6		4.34	2.26, 2.20	1.95, 1.90	3.71, 3.69
N7	8.48	4.64	2.74, 2.66		
V8	8.01	4.01	1.98	0.84	
D9	8.6	4.85	2.79, 2.59		
P10		3.85	2.09, 2.03	1.89, 1.76	2.27, 2.23
N11	8.42		2.79, 2.65		
A12	7.86	4.18	1.32		
N13	8.48		2.74, 2.66		
P14		3.52	2.06, 2.03	1.89, 1.75	2.29, 2.22
N15	8.51	4.66	2.77, 2.68		
V16	7.97	4.04	2.06	0.88	

Description: The data provided represents the assignments of the proton signals derivated form 3.

**Figure 1:**
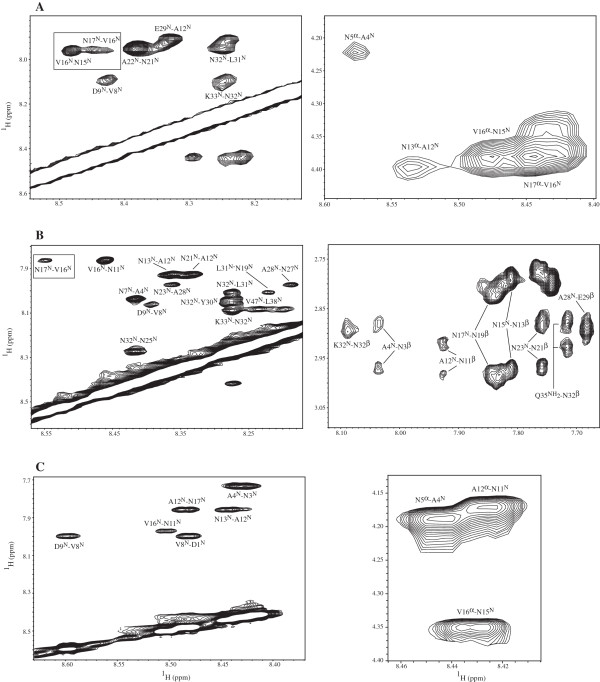
**NOESY spectra collected for 1, 2, and 3.** Portions of the 900 MHz NOESY spectra for **A 1**, **B 2** and **C 3**. **1** and **2** were dissolved in 25% deuterated acetonitrile and 75% H_2_O to a final concentration 1 mM. **3** was dissolved in 10% D_2_O/90% H_2_O to a final concentration 1 mM. The mixing time was 200–250 ms. The left panel displays NOEs in the NH region for each peptide. The left panel shows other structurally significant NOEs detected for these peptides. Signals enclosed in squares are interepitope NOEs.

**Figure 2:**
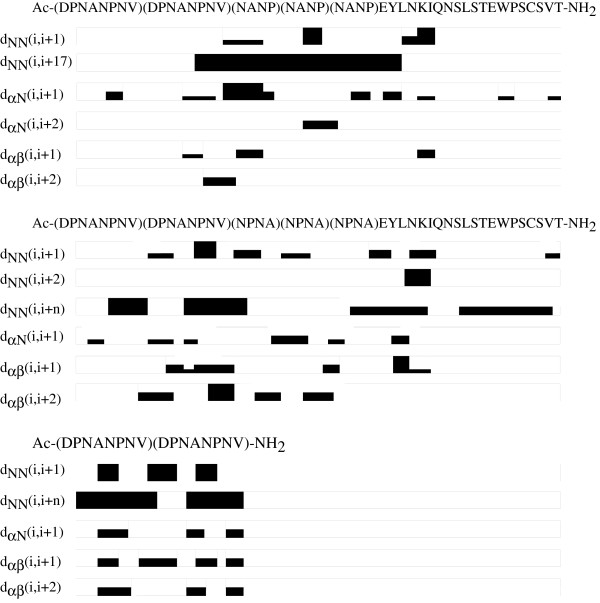
**Summary of NOE connectivities observed for all peptides at 278K.** Data for **1**, **2** and **3** were obtained from 900 MHz NOESY spectra shown in Figure 1.

Portions of the NOESY spectra for 1, 2 and 3 are shown in Figure 1, and the NOE connectivities observed for all peptides are summarized in Figure 2. For peptide 1 the presence of a strong d_NN_(i,i+1) and various medium d_αN_(i,i+1) and d_αN_(i,i+2) NOEs in the central region, B-epitope, indicates that this region of the peptide tends to form turn-like structures as previously indicated by Dyson *et al.*[9]. The right flank of this peptide, T* epitope, displays one strong d_NN_(i,i+1) NOE, with medium d_αβ_(i,i+1), and weak d_αN_(i,i+1). The left flank of 1 exhibits weak d_NN_(i,i+1) and medium and weak d_αN_(i,i+1) and d_αβ_(i,i+2). Both sets of NOEs are indicative of turn-like structures also for the flanks. It is worth noticing that most of the NOEs detected for the T1 and T* epitopes are concentrated towards the ends close to the B epitope. The N- and C- termini of 1 are apparently less structured than its central part.

Helical conformations are hinted in 2 by: 1) a series of strong d_NN_(i,i+1), d_NN_(i,i+3), d_NN_(i,i+5), medium d_NN_(i,i+7) and d_NN_(i,i+9) NOE connectivities in segments A4-N7, N11-V16, N25-N32 and S37-V46; with no d_αN_(i,i+1) connectivities [18]; and, 2) a series of strong and medium d_αβ_(i,i+1) and d_αβ_(i,i+2) [19]. Peptide 3 shows an NOE patter similar to the one exhibited by the same epitope in 2. There are strong long-range d_NN_(i,i+5) and d_NN_(i,i+7) NOE connectivities in segments D1-V8 and N11-V16. Additionally, 3 shows strong d_NN_(i,i+1) NOEs and series of medium d_αβ_(i,i+1) and d_αβ_(i,i+2) NOEs, indicating the presence of a helix-type structure in this peptide also.

The regions where the flanks T1 and T* connect with the central B and B’ epitopes, interepitope regions, display sets of NOE connectivities which are different in both peptides. For 1, there are strong d_αN_(i,i+1) NOE connectivities in segment V16-A18, and medium d_αN_(i,i+1) in segment P28-E29. Additionally, weak d_NN_(16,17) and medium d_αβ_(16,17) NOEs are also present. For 2**,** only a medium d_NN_(16,17) is detected.

### Average structures

**Figure 3:**
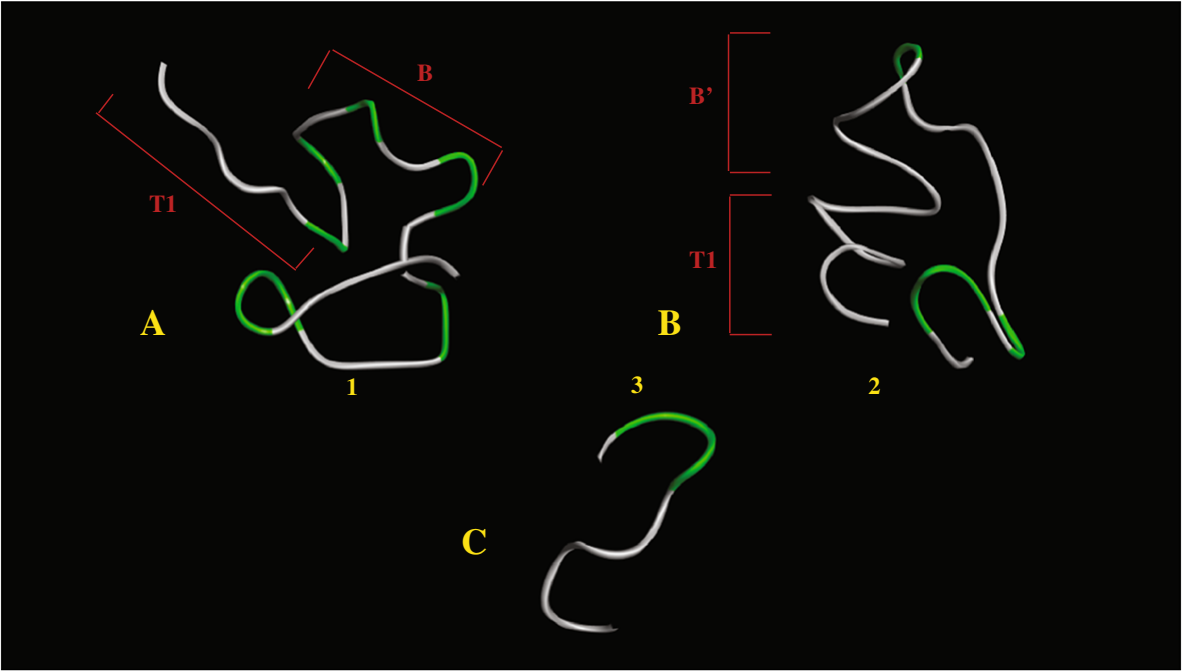
**Calculated structures.** Calculated average structures for A **1**, B **2**, and C **3**. The T1, B and B’ epitope regions are indicated. Peptide **3** is a T1 epitope.

The average structures of 1 and 2 were derived from the NMR data (NOESY) collected for these peptides, and are shown in Figure 3. The molecular dynamic simulations, with NOE-derived distance restrains, show an 85% agreement with the experimental NOE data. These structures indicate that 1 exhibits a β-turn type of structure while 2 adopts a more helical conformation.

### CD spectroscopy

**Figure 4:**
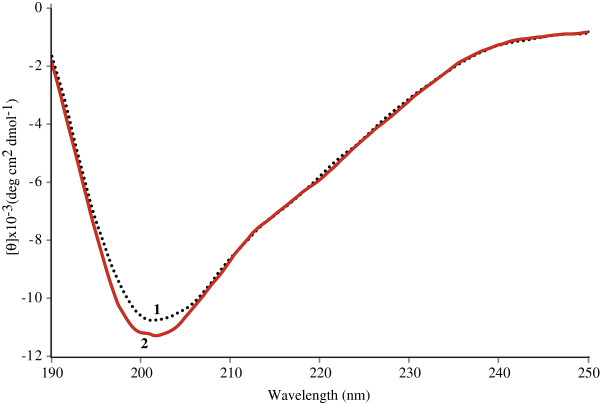
**Circular dichroism spectra. 1** (black) and **2** (red). The solutions were 20 μM in water at the room temperature.

CD spectra for 1 and 2 were collected to confirm the structural preferences of these peptides, proposed based on NMR data (Figure 4). Both peptides showed similar elipticity in the CD spectra, indicating the presence of β-turns.

## Discussion

Evidence supporting structural differences between the NANP and NPNA motives in the CSP have been widely documented in the literature since the publication of the classic 1990 ^1^H NMR study by Dyson *et al.*, revealing that the structural motive of the central region of the CSP corresponded more to the NPNA cadence than to the commonly quoted NANP [13]. The present investigation explores the structural impact that the NPNA sequence has on a proven anti-plasmodial peptide [8], through the structural investigation of the peptide constructs T1BT* and T1B’T*. The NMR results presented herein indicate that the T1BT* peptide, containing the NANP cadence, exhibits a structure radically different from that of its T1B’T* analogue with a modified (NPNA)_3_ B-cell epitope. The NOE evidence strongly suggests that the structured conformers of 1 and 2 contain helical and/or reverse turns, with 2 exhibiting the most helical character. Regarding the CD data, a significant minimum at ~ 222 nm is expected when helical structures are present in solution. The CD spectra in Figure 4 indicate that 2 exhibits a hint of a minimum at ~ 222 nm, while this minimum is absent in the CD spectrum of 1. These facts are consistent with the NMR data collected for 1, and 2, which indicates a more helical character for 2. The lack of a significant minimum in the CD spectrum collected for 2 is not necessarily an indication of the absence of helixes in its structure. In helical peptides where the carbonyl groups are not strictly aligned, CD spectra can exhibit a greatly diminished intensity at 208 and 222 nm [19,20]. The elipticity minimum at ~202 nm, the lack of a significant minimum at 222 nm, and the NMR data for this peptide indicate the that the structural model for 2 should be one where only a small population of molecules contain more than one helical turn [13].

Other NMR data have been used to give information on the conformational preferences of peptides. These include temperature coefficients of the amide proton chemical shift and the ^3^J_HNα_ coupling constant [12,13]. Unfortunately, the presence of repeating units and amino acid duplication in the primary structures of 1 and 2 caused severe overlap of these signals, which prevented the collection this kind of NMR data. In order to further the available knowledge regarding the solution structures of the peptides studied in this investigation, structural explorations with restrained molecular dynamics simulations were performed. The conformational ensemble of most short linear peptides in aqueous solution consists of a large number of rapidly interconverting conformers. The peptide samples a wide variety of conformational states, which makes quantitative calculations of structure meaningful mostly for peptides that adopt unique conformations in solution. However, the average structures calculated in this work can be used to visualize the structural trends depicted by the NMR data, as long as the derived structures are not interpreted as unique. The average structures calculated for 1 and 2 are shown in Figure 3. As mentioned in the results section, the NMR data collected for 2 hints the presence of helical structures, while 1 exhibits a more turn-like conformation. The NMR data derived for 3 also indicates the presence of helical structures. The NMR findings are confirmed by the calculated average structures.

**Figure 5:**
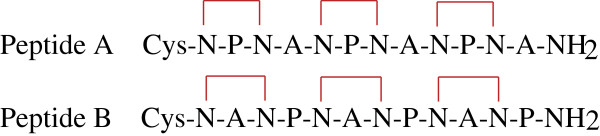
**Conformationally restricted CSP central repeats.** The cross bars represent the hydrogen bonds replaced with ethylene bridges that link N sidechains.

While 1 and 2 only differ from each other in the B epitope, the NMR data collected and the calculated average structures for these peptides are very different from each other. It is safe to infer then that the central B epitope has a strong influence in the global structure of both peptides. The interepitope NOEs detected for 1 and 2 attest for these differences. As mentioned previously, only 1 displays multiple interepitope NOE connectivities. Most of the T1-B interepitope NOEs observed for 1 involve the amino acid sequence N15V16N17A18. The equivalent amino acid sequence in 2 is N15V16N17P18. It appears that the substitution of A18 in 1 with P18 in 2 might change the conformation of the interepitope region. An alternative way of accounting for the structural differences observed between 1 and 2 is to consider the sequences N13P14N15V16N17P18N19A20N21P22N23A24N25P26N27A28 for 2, and N13P14N15V16N17A18N19P20N21A22N23P24N25A26N27P28, for 1. In 2, the T1-epitope region involved in interepitope NOEs, N13P14N15V, is followed by the structural cadence (NPNA)_3_. In 1, the structural cadence is shifted by the presence of the amino acids N17A18, and interrupted at P28. These interferences in the structural cadence alter its original helical conformation, generating the structures depicted in Figure 3A. It is interesting to notice that the helical tendencies of 3 are carried over to 2, but not to 1, which may be a consequence of the different conformational tendencies displayed by both epitopes B and B’. The uninterrupted helical conformations of epitopes T1 and B’ in 2 allow the formation of a continuous helix involving both epitopes, which exhibit mostly intraepitope NOEs. Peptide 2 is possibly an example where cooperative effects leading to a global near-helical conformation for the T1B’T* construct are active. Support of the helical nature of the repeat region of CSP has been provided by structural studies performed by other scientist. The crystal structure of the NPNA peptide was determined [21] and compared to the structure of the NP^Me^NA motif in (NP^Me^NA)_n_ peptides derived from NMR studies [22]. Strong similarities between both molecules were found, indicating a type-I β-turn structure. The lack of full elipticity for NPNA in its crystal structure could be attributed to the presence of only one repeat motif in this peptide. The structure of CSP was determined by Plassmeyer *et al.*[23] through the use of CD, AFM, and molecular modeling. The results of this investigation indicated that the repeat region in CSP forms a stem-like superhelix. Although helical tendencies have not been established as a common trend present in all immunogenic peptides [24], strong correlations of immunogenicity and antigenicity with helix formation have been previously documented [10,25-28]. Despite the variations in structural tendencies observed for immunogenic peptides, which illustrate the diversity of the immune system, it is possible that peptides derived from the CSP are required to have helical structures to interact efficiently with their corresponding antibodies. In that case, a vaccine based NPNA repeat should show higher efficiency as a pre-erythrocyte vaccine that would prevent infection of hepatocytes by sporozoites. Attempts along this line have been reported [29-31]. In an effort to reduce the number of conformations available to native peptides containing the (NANP)_3_ and (NPNA)_3_ sequences, and therefore improve their immunogenicity against live *P. falciparum* sporozoites, Satterthwait and co-workers synthesized conformationally-restrained versions of these peptides where possible hydrogen bonds linking the N residues were replaced by covalent bonds (Figure 5). Among the results derived from these interesting studies, it was observed that peptide A antiserum showed little or no reaction with peptide B. This finding indicates that anti-peptide A antibodies are conformationally specific, and reinforce the notion that structural considerations cannot be put aside when it comes to peptide-based vaccines. Peptide A antiserum also exhibited a strong reaction when titrated against living sporozoites in these studies, indicating that this shaped peptide can generate antiserum that cross reacts with the native form the *P. falciparum* CSP. This result suggests that the NPNA sequence must be the one to deserve a very close look as an integral part of an anti-plasmodial vaccine, rather than its analog NANP.

In the work described herein, the T* epitope only displays important NOE information in the B-T* interepitope region. The average structure calculated for **2** exhibits a mixed structural character to the T* epitope, with helical character close to the B epitope, and a more extended nature toward the C-terminus of the peptide (Figure 3). The crystal structure for region III (amino acids 310 to 375) of CSP was solved by Doud *et al.*[32]. In this structure the T* epitope (amino acids 318 to 337) is distributed among a α1-helix (partial sequence EYLNKIQN-), the linker (partial sequence –SLS-), and the strand 1 (partial sequence –TEWSPCSVT) regions. The structural results are somewhat consistent with the crystal structure of T*, assigning helical character to the first few amino acids in T*, and a more extended tendency for the rest of the peptide. As found for epitope T1, the structural features displayed by T* in 2 are not mirrored by those exhibited by this epitope in 1, since T* loses its helical nature in this construct. This result seems to indicate that having the proper repeat unit in a peptide construct generates cooperative effects that favor structural tendencies toward that of the cognate sequence.

It is well known that the repeat regions of CSPs form different *Plasmodium* exhibit major and minor repeats. In *P. falciparum* CSPs the NPNA and NVDP sequences account for the former and latter, respectively. In spite of their ubiquity in CSPs, the role of the minor repeats has not received enough attention in the investigation of peptide-based vaccines. An extensive comparison of CSP amino acid sequences from various *Plasmodium* including *falciparum, knowlesi, malariae, brasilianum, cynomogli, vivax* and *simium* Vk210 and Vk247, has unveiled the presence of key amino acids in the minor repeats that could play a very important role in the stabilization and cohesion of the overall protein structure. CSP-derived peptides containing minor-repeat elements are currently being characterized to test this hypothesis. If confirmed, the overall helical character of the central region of CSP would be retained, with the minor repeats serving the purpose of determining the relative location of different helical segments.

Another issue that requires close attention when considering peptide-based vaccines is the polymorphism observed for particular epitopes. Among these epitopes T* exhibits a high degree of polymorphism identified to alter the amino acid sequence in the region between E29 and L38 for different *P. falciparum* strains. The elegant studies reported by Parra-López and co-workers on the evaluation of the specificity of the T* sequence regarding its binding to the human class II MHC protein DR4 (HLA-DRB1*041) [33] indicate that the residues in the peptide required for anchoring to DR4; L31, I34, N36, and S39 in the sequence numbering used here; were highly conserved in the *Plasmodium* sequences described to date. On the other hand, our NMR study of peptide constructs containing the T* epitope indicates that it exhibits partial helical character when bound to B’, but it loses it when bound to B. If the following factors are considered: 1) the important protein binding sites are conserved in most strains [33], 2) the required partial elipticity is brought about by cooperative effects (B’ instead of B) as suggested by the results reported herein, and 3) individuals vaccinated with one T* sequence exhibit significant cross-reactivity with variants of the CSP present in other *Plasmodium* strains [34,35]; it can safely be expected that T* polymorphism should not affect the efficacy of malaria vaccines containing it, regardless of the amino acid sequence considered.

The results derived from the present investigation should be helpful in the design of more effective, conformationally defined malaria vaccine candidates. The relative efficiency of T1BT* and T1B’T* as immunogenic peptides remains to be tested. Efforts along this line are underway and will be reported as soon as available.

## Conclusions

The present study investigated the structural behaviour of the synthetic peptide constructs T1BT* and T1B’T*, containing the tandemly repeated motifs (NANP)_3_ and (NPNA), in water-based solvents. Experimental NMR and CD data, in conjunction with molecular modelling, provide additional information about the topology of each peptide, and appears to be a useful tool to design efficient vaccines that might be used against various *Plasmodium* species. The presence of B’ in in T1B’T* seems to stimulate cooperative effects, which help shape epitopes T1 and T*in conformations similar to those assumed by these peptides in the cognate structure of CSP. The work presented also highlights the importance of the structural aspects of biological molecules when it comes to consider them as vaccine candidates. Antigen-antibody interactions require the proper structural conformations to be effective. The structural contributions of both members of this medicinal couple cannot be ignored, and a lot of trial-and-error work could be avoided, if the structures of chemotherapeutic molecules are considered in detail as part of the research performed around them.

## Abbreviations

CSP: Circumsporozoite protein; CD: Circular dichroism; CW: Continuous wave; FMOC: Fluorenylmethyloxycarbonyl; HPLC: High-performance liquid chromatography; NOE: Nuclear Overhauser effect cross signal; NOESY: Nuclear Overhauser effect spectroscopy; ps: Picosecond; rms: Root-mean-square deviation; TOCSY: Totally correlated spectroscopy

## Competing interests

The authors declare that they have no competing interests.

## Authors’ contributions

ET prepared the NMR samples, collected NMR data at 600 MHz, analysed NMR data collected at 900 and 600 MHz, performed the molecular dynamics calculations, and contributed to the draft paper. GA collected and analysed data at 900 MHz. KB analysed data collected at 600 and 900 MHz. GB and JK synthesized the single-labelled peptides. TL was principal investigator. All authors read and approved the final manuscript.
